# Research and application of digital technology in the diagnosis and treatment of androgenetic alopecia

**DOI:** 10.3389/fdgth.2026.1854199

**Published:** 2026-08-06

**Authors:** Xi Wei, Jingjing Wang, Yicheng Zhang, Xiaojun Yan, Yating Xiao, Yutong Ci, Xiaoling Luo, Xue Wang

**Affiliations:** 1 Department of Dermatology, The First Affiliated Hospital of Shihezi University, Shihezi, Xinjiang, China; 2 School of Medicine, Shihezi University, Shihezi, Xinjiang, China; 3 Department of Ultrasound Medicine, The First Affiliated Hospital of Shihezi University, Shihezi, Xinjiang, China; 4 Library, Huazhong University of Science and Technology, Wuhan, Hubei, China; 5 Subject Service Center, The Library of Shihezi University, Shihezi, Xinjiang, China

**Keywords:** androgenetic alopecia, artificial intelligence-assisted diagnosis, clinical practice, digital technology, telemedicine

## Abstract

Androgenetic alopecia (AGA) is the most common type of alopecia in the world. Although AGA may not cause physical damage to the human body, it will bring serious psychological harm and a decline in quality of life. In the past, the diagnosis and treatment of AGA in clinic mostly depended on the experience of doctors. The introduction of digital technology will provide visual images for the hair characterization of AGA, assist the staging evaluation and differential diagnosis of AGA, and shows potential for improving efficiency and accuracy in reported studies under specific conditions. In terms of treatment, laser treatment is an important means of AGA; telemedicine makes the treatment and nursing of AGA more convenient and quick; at the same time, the application of digitization also helps to optimize AGA efficacy evaluation, treatment strategies, and new drug development. In order to further summarize some new diagnosis and treatment ideas and schemes in clinical practice, this paper deeply studies and analyzes the application of digital technology in the diagnosis and treatment of AGA in recent years, aiming to provide a more efficient, convenient and personalized scheme for clinical practice.

## 1 Introduction

Androgenetic alopecia (AGA), also known as pattern baldness, is a progressive hair loss disorder that most commonly occurs after puberty (1). In males, it primarily manifests as a receding hairline at the forehead and/or progressive thinning and reduction of hair density at the crown region (2, 3). In women, it primarily manifests as progressive thinning and reduction of hair, predominantly on the crown area, generally without a receding frontal hairline. It is referred to as female pattern hair loss (FPHL) (4). A minority of cases may involve the temporal regions, or even diffuse involvement of the entire scalp (4, 5). Androgens and genetic predisposition are key mechanisms in the pathogenesis of AGA (6, 7). The prevalence of this condition varies significantly across ethnic groups, being higher in Caucasians and lower in African Americans and Asians (8). In China, the prevalence rate among adult males is 21.3%, while that among adult females is 6.0% (9).

Although AGA does not cause physical harm to human health, it can impose significant psychosocial burdens on patients. As their appearance changes, patients experience reduced self-confidence, diminished satisfaction with their physical image, and a decline in quality of life (10, 11). At the same time, people tend to hold negative perceptions toward AGA patients, often avoiding interaction or employment opportunities with them (10). This significantly impacts patients’ ability to work and live normally, while also strengthening their motivation to seek treatment (12). Additionally, due to increased pressures in work, daily life, and education within contemporary society, AGA is showing a trend toward affecting younger individuals (13, 14).

Digital technologies in digital medicine refer to a technical system that employs computer science, artificial intelligence (AI), big data, telemedicine, automation, and other technological means to collect, analyze, and apply medical data, thereby enhancing the efficiency and precision of diagnosis and treatment (15, 16). The diagnosis of AGA traditionally relies on clinical examination and physician experience, which involves significant subjectivity and lacks objective, standardized approaches. With the advancement of digital technology and AI, their integration into AGA clinical management has emerged as a research hotspot in recent years. In reported studies under specific conditions, applications such as digital image analysis, high-precision diagnostic grading models based on deep learning, and telemedicine hold considerable potential, offering possibilities for rapid clinical diagnosis and quantitative assessment of AGA (17–21). This paper summarizes recent reports on the clinical application of digital technologies in AGA.

This narrative review was conducted following a comprehensive literature search strategy. We systematically searched PubMed, Web of Science, Embase, and Cochrane Library databases from January 2010 to December 2025. The search terms included: “androgenetic alopecia,” “digital technology,” “artificial intelligence,” “machine learning,” “deep learning,” “telemedicine,” “trichoscopy,” “laser therapy,” and “hair loss treatment.” Inclusion criteria were: (1) peer-reviewed articles published in English; (2) studies focusing on digital technology applications in AGA diagnosis or treatment; (3) clinical studies, randomized controlled trials, systematic reviews, and meta-analyses. Exclusion criteria were: (1) case reports with less than 10 patients; (2) conference abstracts without full-text availability; (3) studies not specifically related to AGA (22).

## 2 Application of digital technology in the diagnosis of androgenetic alopecia

### 2.1 Applications of the trichoscope

Androgenetic alopecia is the most common form of hair loss, but classification systems for grading AGA remain limited. Commonly used systems include the male Hamilton-Norwood classification and the female three-stage (Ludwig) classification system (23). The diagnosis of AGA is typically based on clinical presentation, and analysis of hair patterns helps dermatologists better assess disease activity (24). To avoid invasive diagnostic methods such as scalp biopsies, non-invasive trichoscopy has become an important diagnostic tool (25).

Trichoscopy is a novel non-invasive diagnostic technique that magnifies hair shaft images 20 to 1000 times. Based on distinct microscopic characteristics, it aids in differentiating non-scarring hair loss conditions such as androgenetic alopecia, alopecia areata, trichotillomania, and telogen effluvium (26). Trichoscopy can reveal changes in follicular diameter during the early clinical stages and can examine large areas within a short timeframe. It provides observable information about the hair shaft, follicular openings, perifollicular and interfollicular skin surfaces, and cutaneous vessels in AGA, presenting easily identifiable images. In reported studies, it shows promise as a potential tool for aiding diagnosis (27, 28). Follicle mapping technology identifies follicle opening coordinates in trichoscopic images through pattern recognition, generating unique scalp region identifiers for precise follow-up localization (29). Additionally, simple digital recording fulfills the need for both physicians and patients to simultaneously view video-graphic images, enhancing the accuracy of AGA diagnosis and treatment by comparing pre- and post-treatment images. Based on these characteristics, trichoscopy may assist in measuring and evaluating disease activity, severity, and prognosis, as well as potentially supporting adjustments to therapeutic medication (27, 30, 31).

### 2.2 AI-Assisted hair mirror image analysis

Traditional trichoscopes rely on visual assessment by physicians, but AI models enable automated counting and feature extraction. AI programs developed in recent years assist trichoscopes in automatically calculating data such as hair density, hair diameter, and pigmentation. Under specific experimental conditions, it helps mitigate the limitations of trichoscopes being highly dependent on visual observation, potentially reduces subjective human error, and provides relatively precise quantification within each field of view (32, 33).

Currently, AGA staging remains based on macroscopic scales, and the classification results for fundamental and specific patterns of male androgenetic alopecia are subjective. Hair data, such as hair density and diameter distribution observed through trichoscopy, represent potential quantitative indicators. Therefore, Gao et al. developed a deep learning framework for automatically analyzing trichoscopy images and a quantitative model for predicting fundamental and specific classifications in male androgenetic alopecia. The study collected a total of 2,910 trichoscopic images. Leveraging hair data provided by a Convolutional Neural Network (CNN) framework, it accurately analyzes hair density and hair diameter distribution with a detection time of approximately 2 s (17). Katoulis et al. designed a novel three-step algorithm integrating clinical and trichoscopic hair features for pattern analysis and diagnosis, significantly reducing the need for invasive diagnostic procedures (34).

Di Fraia et al. employed Support Vector Machine (SVM) models and AI assistance to perform automated counting of trichoscopic patterns. Using Trichoscale Pro software, they photographed trichoscopic images from 200 AGA patients and conducted trichoscopic analysis. They quantified the number of vellus hairs, empty follicles, and hair follicle units within each photograph to assess the severity of AGA and assist in grading decisions (18).

Notably, the trichoscopic images used in these studies were collected and annotated under the supervision of board-certified dermatologists with expertise in hair disorders. In Gao et al.'s study, all image annotations were independently validated by two experienced dermatologists, and disagreements were resolved through consensus discussion with a senior trichologist (17). Similarly, in Di Fraia et al.'s research, the ground-truth labels for AGA severity grading were established by expert dermatologists specializing in trichoscopy, ensuring that the AI-generated classifications were validated against clinically authoritative assessments (18). These multidisciplinary collaborations between computer scientists and dermatologists provide essential clinical credibility to the computational findings.

### 2.3 Artificial intelligence-assisted diagnosis system

AI applications or models hold preliminary potential for detection and auxiliary diagnosis in the field of skin and hair conditions. Larger databases can enhance the accuracy and predictive capabilities of these AI applications or models. Tibot is an AI application that analyzes uploaded images of skin and hair conditions and provides answers to user inquiries. Comparative studies have demonstrated that AI diagnostic applications show promising accuracy and sensitivity under specific conditions, which may help address the diagnostic needs of some AGA patients (35, 36). Additionally, AI-assisted dermatoscopy aids in the clinical assessment of AGA classification and severity grading, with potential to enhance diagnostic accuracy and efficiency (pending further clinical validation). The convolutional neural network designed by Gao et al. achieved over 90% accuracy in predicting AGA, while Di Fraia et al.'s SVM model demonstrated 94.3% accuracy on the training dataset and 90.0% accuracy on the test dataset, both demonstrating preliminary potential in their respective study populations (17, 18). A medical team from South Korea has developed and validated a deep learning model for the automated early detection of AGA using trichoscopic images. This model demonstrates diagnostic accuracy exceeding 90%, exhibiting high precision and universality, and holds potential application value as a clinical and teledermatology screening tool (19). In the quantitative assessment of scalp lesions, artificial intelligence algorithms can automate the Dermascope scoring system, which traditionally requires trained evaluators to grade scalp characteristics. By leveraging hair mirror image recognition, AI achieves standardized and repeatable evaluations of scalp conditions (37, 38). These AI tools have been clinically validated for screening high-risk populations, assisting dermatologists in busy clinical settings, and providing preliminary assessments in resource-limited areas where specialist access is restricted (17, 18, 39). However, it should be noted that these accuracy rates were primarily derived from internal validation datasets, and external validation in diverse clinical populations remains limited (40).

It is important to emphasize that the clinical validation of these AI systems has been consistently conducted by or in collaboration with dermatologists. For instance, the Tibot application was evaluated through prospective studies in which board-certified dermatologists provided the reference standard diagnoses against which AI predictions were compared (35, 36). In the study by Suh et al., a team of dermatologists from a tertiary hospital in South Korea manually reviewed and labeled all trichoscopic images to establish the ground-truth dataset, and the final AI model performance was independently verified by blinded dermatologist assessment (19). Furthermore, the development of the SVM model by Di Fraia et al. involved direct participation of dermatology clinicians in feature selection and model validation, ensuring that the computational outputs aligned with real-world clinical interpretations (18). These expert-validated approaches confirm that the computer-generated data in AGA diagnosis have been appropriately authenticated by qualified dermatology specialists.

AI applications not only assist in diagnosing AGA (17, 19, 37) but also offer features such as determining whether and when to see a doctor, scheduling appointments, ordering medications and skincare products, consulting dermatologists online, providing reminders for treatment dates and appointments, and offering a dermatology dictionary explaining causes, symptoms, and treatments (41–44). Artificial intelligence and its applications may become a significant aid for healthcare professionals in image acquisition, processing, interpretation, reporting, and follow-up planning, while also supporting personalized treatment for AGA patients (20, 45–48).

## 3 Application of digital technology in the treatment of androgenetic alopecia

### 3.1 Laser therapy

Laser therapy for hair loss has gained significant popularity in recent years and is promoted for its effectiveness in preventing hair loss. The most widely recognized and safe treatment methods include low-level laser therapy (LLLT) and non-ablative erbium lasers, such as the HairMax Lasercomb and laser hair growth caps (49, 50). The biological mechanism remains unclear, but current theories suggest laser therapy alters molecular signaling, cytokines, and hair growth genes within the hair follicle growth cycle, inducing a faster transition from the resting phase to the growth phase (51, 52). Among the existing clinical evidence for laser therapy in AGA, only a minority of studies are randomized controlled trials (RCTs), such as those by Faghihi et al. (53) and Suchonwanit et al. (54); the remaining citations are mostly systematic reviews or observational studies (49, 50, 55, 56). Multiple studies indicate that laser therapy significantly improves hair diameter and count. Furthermore, when combined with treatments like platelet-rich plasma (PRP) or minoxidil for androgenetic alopecia (AGA), the combined therapy yields superior results compared to monotherapy (53–56).

Additionally, laser-assisted techniques for drug delivery represent an emerging technology with broad potential clinical applications. Lasers can facilitate drug delivery through laser channels, tissue ablation, light energy conversion, or non-ablative superficial resurfacing (57). Among these, one RCT has compared the efficacy and safety of fractional erbium glass laser combined with topical 5% minoxidil vs. 5% minoxidil alone for treating male AGA. Results indicate superior outcomes with the former approach (54). However, high-quality RCTs supporting laser therapy for AGA remain relatively limited, and existing studies exhibit heterogeneity in sample size, follow-up duration, and laser parameters, and substantial additional research data are still required to determine whether laser therapy can be incorporated into the treatment algorithm for hair loss (52, 56).

In addition to non-invasive laser and microneedling treatments, AGA non-pharmaceutical therapies also include hair restoration surgery, which employs robotic technology for follicular unit extraction and separation (58).

### 3.2 Digital technologies in therapeutic efficacy evaluation

Artificial intelligence in preliminary explorations has shown potential to standardize hair loss treatment evaluation in clinical research. Current methods for assessing treatment efficacy include holistic photo comparisons, manual trichoscopy examinations, physician subjective assessments, and patient self-reports—all qualitative and subjective approaches. AI algorithms enable quantitative analysis of three-dimensional hair bundle characteristics (such as local/global hair density, arrangement regularity, and frizziness), achieving objective quantitative evaluation (59). In a study, Raymond et al. employed AI photography during clinical practice to collect automated quantitative and qualitative data on hair density following PRP therapy and scalp health. Utilizing AI devices, they obtained hair fiber density measurements, documenting early treatment trends for PRP therapy in AGA. This approach offers a potentially more quantitative reference method for evaluating clinical efficacy (60). Researchers have also leveraged ChatGPT's visual assessment capabilities to statistically analyze the visible conditions in each scalp image for efficacy evaluation (61). Additionally, machine learning can predict hair growth trends in FPHL and determine its clinical characteristics, thereby optimizing efficacy assessment and personalized treatment for FPHL patients (62).

Deep learning (DL) combined with optical coherence tomography (OCT) enables non-invasive assessment of hair and follicle counts, thereby facilitating more accurate and effective monitoring of treatment efficacy. Additionally, OCT captures a scalp region in just 3 s, while the DL model delivers predictions in 1 s with an error rate below 20% (21). Compared to traditional methods without digital assistance, this emerging approach requires only half a minute. Thus, it in specific clinical settings may help save clinicians’ time and effort compared to visual inspection, with potential to become a candidate non-invasive standard for evaluating AGA treatment progress (21).

However, it should be noted that the studies cited in this section primarily consist of proof-of-concept studies, case series, or exploratory technical methodologies (21, 59–62), rather than randomized controlled trials; thus, the efficacy of AI-based assessment methods in AGA treatment still requires validation through RCTs.

### 3.3 Telemedicine

Although AGA is a benign condition, it significantly impairs patients’ quality of life. An increasing number of men experiencing hair loss worry about the negative impacts on their self-esteem and perceived attractiveness (10). Consequently, even though AGA does not cause physical harm, patients’ willingness to seek treatment is steadily rising (42). However, within traditional medical settings, approximately one-fifth of patients feel uncomfortable discussing hair loss issues. Over half believe they did not receive effective treatment recommendations and lose interest in pursuing treatment after consulting a physician (42). Compared to traditional healthcare, dermatology patients demonstrate a strong interest in digital health services (42, 43). Direct-to-consumer teledermatology services, such as online prescription platforms (OPPs), have seen increased adoption for AGA telemedicine diagnosis and treatment in recent years (42).It is noteworthy that the qualifications of medical personnel vary across different OPPs, and some platforms may not be operated by board-certified dermatologists with expertise in hair disorders (44). Most patients access these platforms via mobile devices (63, 64). OPPs offer round-the-clock accessibility, allowing users to upload lesion images and complete questionnaires to obtain diagnoses and prescriptions (42, 65, 66). This eliminates geographical constraints tied to hospital locations, significantly enhancing convenience. This advantage contrasts sharply with traditional care models. In principle, teledermatology reduces geographic barriers, enabling patients outside urban areas to access treatment and has the potential to promote equitable distribution of healthcare resources (42). Additional research indicates that younger physicians exhibit higher adoption rates of telemedicine (66, 67). An increasing number of young people are choosing telemedicine for dermatological consultations, reflecting a trend toward younger demographics among both healthcare providers and patients (68).

Furthermore, teledermatology under specific conditions shows potential as an auxiliary tool for treating patients with hair loss. Patients managed through digital health platforms in some observational studies have demonstrated relatively higher medication adherence and reported relatively better treatment outcomes (69, 70). Simultaneously, these platforms are advancing public health education and awareness, leveraging the internet's advantages to make digital care accessible to more people, thereby enhancing overall health levels (43).

Combined with remote trichoscopy, telemedicine services not only diagnose hair disorders but also enhance follow-up efficiency, increase patient compliance, and improve patient satisfaction with care (48, 69). Dermatologists can also use telemedicine to guide patients in adjusting oral medications like minoxidil, implement effective systems for managing follow-ups of patients with chronic recurrent scalp conditions, and maintain continuity of care (48). However, teledermatology consultations for hair loss currently lag far behind other skin conditions (20). More telemedicine guidelines should be developed to leverage telemedicine's advantages, facilitating treatment monitoring, personalized care, and follow-up for hair loss patients (20).

Current evidence supporting the use of telemedicine for AGA management primarily derives from observational studies, cross-sectional surveys, and retrospective analyses (42, 43, 63–70), with no randomized controlled trials reported to date. Future research requires RCTs to compare the efficacy and safety of telemedicine vs. traditional in-person consultation models in AGA patients.

Telemedicine, backed by internet platforms and big data, offers multiple advantages: it reduces patients’ medical expenses, facilitates convenient access to care, and enhances healthcare accessibility and equity (71, 72). However, due to unresolved legal, ethical, privacy, and liability issues surrounding AI-based diagnostics, it faces challenges in obtaining regulatory approval from relevant authorities (45, 47, 73). Additionally, issues such as the inability to conduct excessive additional testing for prescription abuse and the lack of thorough qualification verification by online medical personnel also hinder the development of telemedicine (44, 67, 74). To develop high-quality applications, experts from computer science, biomedicine, and medical fields must still be involved in the process (46).

## 4 Application of digital technology in research and development related to androgenetic alopecia

Oxidative stress is considered one of the important mechanisms involved in AGA pathogenesis. Targeting this mechanism, Zhang et al. successfully designed a highly mimetic manganese thiophosphite (MnPS_3_) superoxide dismutase (SOD) analog using machine learning tools; MnPS_3_ can be delivered deep into the skin to hair follicles via a microneedle patch (MnPS_3_ microneedle patch, MnMNP), where it scavenges excess reactive oxygen species. MnMNP demonstrated stronger hair regrowth effects than minoxidil (this finding is based on *in vitro* experiments and animal models; its efficacy and safety in humans require further validation through clinical trials). Applying machine learning-derived materials to SOD-like nanozyme research not only accelerates the screening of such substances but also holds significant implications for rationally designing novel nanozymes (75).

Deep learning models can predict protein structures, perform computational modeling, and forecast spatial interactions between proteins. Consequently, Ma et al. discovered a proteolysis-targeting chimera (PROTAC) drug targeting the androgen receptor. This demonstrates in silico the immense application potential of DL models in advancing new drug discovery for AGA and enabling personalized medicine for AGA (76).

Research also indicates that machine learning-driven therapeutic strategies can optimize the delivery of AGA treatment agents. Direct injection of PRP causes pain, while microneedles (MNs) infused with PRP exhibit low hardness and slow dissolution. To address this challenge, Yan's team proposed a machine learning-driven strategy integrating therapeutic substance selection, orthogonal experimental design, ML prediction, and Pareto frontier identification. This approach identifies optimal material compositions that simultaneously achieve high hardness and rapid dissolution, enhancing patient compliance and comfort. The developed framework shows potential under experimental conditions, but its clinical translation requires validation in human trials; it holds promise as a scalable model to accelerate the clinical translation of biomaterials like MNs (77).

Despite the promising results, current AI models for AGA diagnosis have several important limitations that should be acknowledged. First, most AI models have been trained and validated on datasets from specific ethnic populations, raising concerns about generalizability across diverse patient groups. Second, external validation in independent clinical settings remains limited, with most studies reporting internal validation results only. Third, regulatory barriers exist, as most AI diagnostic tools have not yet received approval from major regulatory bodies such as the FDA or EMA. Fourth, dataset bias is a significant concern, as training data may not adequately represent all AGA subtypes and severity grades. Finally, the “black box” nature of deep learning algorithms poses challenges for clinical interpretability and acceptance. Additionally, the economic viability and cost-effectiveness of these emerging technologies relative to traditional treatments remain insufficiently evaluated, which may constrain their widespread adoption in clinical practice. These limitations highlight the need for more robust, externally validated, and regulatory-compliant AI solutions before widespread clinical adoption (78).

Moreover, although the cited studies involved dermatologists in their validation processes, the extent of expert involvement varied considerably across different investigations. Some studies relied on single-center dermatologist assessments (17, 18), while others employed multicenter expert panels (19). The lack of standardized protocols for dermatologist-AI collaborative validation across institutions represents an additional challenge that needs to be addressed to ensure consistent clinical reliability of computer-generated diagnostic data.

## 5 Conclusions

Digital technologies offer new possibilities for the diagnosis and management of AGA. In diagnosis, trichoscopy and AI-assisted image analysis systems can aid in staging evaluation and differential diagnosis of AGA under specific conditions, with some deep learning models demonstrating high accuracy in internal validation. In treatment, low-level laser therapy and laser-assisted drug delivery have shown potential to improve hair density and diameter; the integration of AI algorithms with optical coherence tomography provides objective quantitative tools for efficacy assessment; and telemedicine offers convenient access to care, with observational studies suggesting improved medication adherence. In research and development, machine learning has facilitated the discovery of novel superoxide dismutase mimetics, proteolysis-targeting chimera drugs, and optimized microneedle delivery systems. Nevertheless, current AI models are largely limited to internal validation in specific populations, with insufficient external generalization and regulatory approval, alongside challenges including dataset bias and algorithmic interpretability. Future multi-center prospective studies and standardized evaluation frameworks are warranted to facilitate the rigorous clinical integration of digital technologies in AGA management.

## References

[1] Kanti V Messenger A Dobos G Reygagne P Finner A Blumeyer A. Evidence-based (S3) guideline for the treatment of androgenetic alopecia in women and in men - short version. J Eur Acad Dermatol Venereol. (2018) 32(1):11–22. 10.1111/jdv.14624 29178529

[2] Sinclair R. Male pattern androgenetic alopecia. Br Med J. (1998) 317(7162):865–9. 10.1136/bmj.317.7162.865 9748188 PMC1113949

[3] Nestor MS Ablon G Gade A Han H Fischer DL. Treatment options for androgenetic alopecia: efficacy, side effects, compliance, financial considerations, and ethics. J Cosmet Dermatol. (2021) 20(12):3759–81. 10.1111/jocd.14537 34741573 PMC9298335

[4] Fabbrocini G Cantelli M Masarà A Annunziata MC Marasca C Cacciapuoti S. Female pattern hair loss: a clinical, pathophysiologic, and therapeutic review. Int J Womens Dermatol. (2018) 4(4):203–11. 10.1016/j.ijwd.2018.05.001 30627618 PMC6322157

[5] Heo JH Yeom SD Byun JW Shin J Choi GS. Significant relationship between temporal hair loss and other scalp areas in female pattern hair loss. J Dermatol. (2020) 47(4):334–41. 10.1111/1346-8138.15220 31919884

[6] Sadasivam IP Sambandam R Kaliyaperumal D Dileep JE. Androgenetic alopecia in men: an update on genetics. Indian J Dermatol. (2024) 69(3):282. 10.4103/ijd.ijd_729_23 39119311 PMC11305502

[7] Chen S Li L Ding W Zhu Y Zhou N. Androgenetic alopecia: an update on pathogenesis and pharmacological treatment. Drug Des Devel Ther. (2025) 19:7349–63. 10.2147/DDDT.S542000 40873858 PMC12380480

[8] Hoffmann R Happle R. Current understanding of androgenetic alopecia. Part II: clinical aspects and treatment. Eur J Dermatol. (2000) 10(5):410–7. 10882953

[9] Wang TL Zhou C Shen YW Wang XY Ding XL Tian S. Prevalence of androgenetic alopecia in China: a community-based study in six cities. Br J Dermatol. (2010) 162(4):843–7. 10.1111/j.1365-2133.2010.09640.x 20105167

[10] Aukerman EL Jafferany M. The psychological consequences of androgenetic alopecia: a systematic review. J Cosmet Dermatol. (2023) 22(1):89–95. 10.1111/jocd.14983 35403805 PMC10084176

[11] Gonul M Cemil BC Ayvaz HH Cankurtaran E Ergin C Gurel MS. Comparison of quality of life in patients with androgenetic alopecia and alopecia areata. An Bras Dermatol. (2018) 93(5):651–8. 10.1590/abd1806-4841.20186131 30156613 PMC6106669

[12] Aksac SE Bilgili SG Yavuz GO Yavuz IH Aksac M Karadag AS. Evaluation of biophysical skin parameters and hair changes in patients with acne vulgaris treated with isotretinoin, and the effect of biotin use on these parameters. Int J Dermatol. (2021) 60(8):980–5. 10.1111/ijd.15485 33682085

[13] Jang WS Son IP Yeo IK Park KY Li K Kim BJ. The annual changes of clinical manifestation of androgenetic alopecia clinic in Korean males and females: a outpatient-based study. Ann Dermatol. (2013) 25(2):181–8. 10.5021/ad.2013.25.2.181 23717009 PMC3662911

[14] Kaya Erdogan H Bulur I Kocaturk E Yildiz B Saracoglu ZN Alatas O. The role of oxidative stress in early-onset androgenetic alopecia. J Cosmet Dermatol. (2017) 16(4):527–30. 10.1111/jocd.12300 27987270

[15] Dunn P Hazzard E. Technology approaches to digital health literacy. Int J Cardiol. (2019) 293:294–6. 10.1016/j.ijcard.2019.06.039 31350037

[16] Cordeiro JV. Digital technologies and data science as health enablers: an outline of appealing promises and compelling ethical, legal, and social challenges. Front Med (Lausanne). (2021) 8:647897. 10.3389/fmed.2021.647897 34307394 PMC8295525

[17] Gao M Wang Y Xu H Xu C Yang X Nie J. Deep learning-based trichoscopic image analysis and quantitative model for predicting basic and specific classification in male androgenetic alopecia. Acta Derm Venereol. (2022) 102:adv00635. 10.2340/actadv.v101.564 34935989 PMC9631273

[18] Di Fraia M Tieghi L Magri F Caro G Michelini S Pellacani G. A machine learning algorithm applied to trichoscopy for androgenic alopecia staging and severity assessment. Dermatol Pract Concept. (2023) 13(3):e2023136. 10.5826/dpc.1303a136 37557111 PMC10412074

[19] Suh MJ Ahn S Byun JY. Automated early detection of androgenetic alopecia using deep learning on trichoscopic images from a Korean cohort: a retrospective model development and validation study. Ewha Med J. (2025) 48(3):e44. 10.12771/emj.2025.00486 40739969 PMC12362284

[20] Bermudez NM Perez S Nguyen B Tosti A. Teledermatology in alopecia: a systematic review. JAAD International. (2024) 14:64–8. 10.1016/j.jdin.2023.10.004 38274395 PMC10809123

[21] Urban G Feil N Csuka E Hashemi K Ekelem C Choi F. Combining deep learning with optical coherence tomography imaging to determine scalp hair and follicle counts. Lasers Surg Med. (2021) 53(1):171–8. 10.1002/lsm.23324 32960994

[22] Moher D Liberati A Tetzlaff J Altman DG Group P. Preferred reporting items for systematic reviews and meta-analyses: the PRISMA statement. PLoS Med. (2009) 6(7):e1000097. 10.1371/journal.pmed.1000097 19621072 PMC2707599

[23] Gupta M Mysore V. Classifications of patterned hair loss: a review. J Cutan Aesthet Surg. (2016) 9(1):3–12. 10.4103/0974-2077.178536 27081243 PMC4812885

[24] Starace M Orlando G Alessandrini A Piraccini BM. Female androgenetic alopecia: an update on diagnosis and management. Am J Clin Dermatol. (2020) 21(1):69–84. 10.1007/s40257-019-00479-x 31677111

[25] Jain N Doshi B Khopkar U. Trichoscopy in alopecias: diagnosis simplified. Int J Trichology. (2013) 5(4):170–8. 10.4103/0974-7753.130385 24778525 PMC3999645

[26] Errichetti E Stinco G. Dermoscopy in general dermatology: a practical overview. Dermatol Ther (Heidelb). (2016) 6(4):471–507. 10.1007/s13555-016-0141-6 27613297 PMC5120630

[27] Kuczara A Waskiel-Burnat A Rakowska A Olszewska M Rudnicka L. Trichoscopy of androgenetic alopecia: a systematic review. J Clin Med. (2024) 13(7):1962. 10.3390/jcm13071962 38610726 PMC11012765

[28] Chiramel MJ Sharma VK Khandpur S Sreenivas V. Relevance of trichoscopy in the differential diagnosis of alopecia: a cross-sectional study from north India. Indian J Dermatol Venereol Leprol. (2016) 82(6):651–8. 10.4103/0378-6323.183636 27297280

[29] Kasprzak M Sicinska J Tosti A. Follicular map: a novel approach to quantitative trichoscopy. Skin Appendage Disord. (2019) 5(4):216–20. 10.1159/000497193 31367599 PMC6615326

[30] Rakowska A Slowinska M Kowalska-Oledzka E Olszewska M Rudnicka L. Dermoscopy in female androgenic alopecia: method standardization and diagnostic criteria. Int J Trichology. (2009) 1(2):123–30. 10.4103/0974-7753.58555 20927234 PMC2938574

[31] Ummiti A Priya PS Chandravathi PL Kumar CS. Correlation of trichoscopic findings in androgenetic alopecia and the disease severity. Int J Trichology. (2019) 11(3):118–22. 10.4103/ijt.ijt_103_17 31360040 PMC6580806

[32] Gupta AK Ivanova IA Renaud HJ. How good is artificial intelligence (AI) at solving hairy problems? A review of AI applications in hair restoration and hair disorders. Dermatol Ther. (2021) 34(2):e14811. 10.1111/dth.14811 33496058

[33] Jartarkar S Patil A Waskiel-Burnat A Rudnicka L Starace M Grabbe S. Artificial intelligence in hair and nail disorders. J Drugs Dermatol. (2022) 21(10):1049–52. 10.36849/JDD.6519 36219051

[34] Katoulis AC Pappa G Sgouros D Markou E Kanelleas A Bozi E. A three-step diagnostic algorithm for alopecia: pattern analysis in trichoscopy. J Clin Med. (2025) 14(4):1195. 10.3390/jcm14041195 40004726 PMC11856343

[35] Patil S Rao ND Patil A Basar F Bate S. Assessment of tibot® artificial intelligence application in prediction of diagnosis in dermatological conditions: results of a single centre study. Indian Dermatol Online J. (2020) 11(6):910–4. 10.4103/idoj.IDOJ_61_20 33344338 PMC7735000

[36] Marri SS Inamadar AC Janagond AB Albadri W. Analyzing the predictability of an artificial intelligence app (tibot) in the diagnosis of dermatological conditions: a cross-sectional study. JMIR Dermatol. (2023) 6:e45529. 10.2196/45529 37632978 PMC10335135

[37] Kim BR Kim MJ Koo J Choi H-J Paik KH Kwon SH. Artificial intelligence-based prescription of personalized scalp cosmetics improved the scalp condition: efficacy results from 100 participants. J Dermatolog Treat. (2024) 35(1):2337908. 10.1080/09546634.2024.2337908 38616301

[38] Chowdhury MS Sultan T Jahan N Mridha MF Safran M Alfarhood S. Leveraging deep neural networks to uncover unprecedented levels of precision in the diagnosis of hair and scalp disorders. Skin Res Technol. (2024) 30(4):e13660. 10.1111/srt.13660 38545843 PMC10974725

[39] Uwishema O Ghezzawi M Charbel N Alawieh S Roy S Wojtara M. Diagnostic performance of artificial intelligence for dermatological conditions: a systematic review focused on low- and middle-income countries to address resource constraints and improve access to specialist care. Int J Emerg Med. (2025) 18(1):172. 10.1186/s12245-025-00975-4 41023774 PMC12481837

[40] Daneshjou R Smith MP Sun MD Rotemberg V Zou J Lack of transparency and potential bias in artificial intelligence data sets and algorithms: a scoping review. JAMA Dermatol. (2021) 157(11):1362–9. 10.1001/jamadermatol.2021.3129 34550305 PMC9379852

[41] Wongvibulsin S Yan MJ Pahalyants V Murphy W Daneshjou R Rotemberg V. Current state of dermatology Mobile applications with artificial intel ligence features. JAMA Dermatol. (2024) 160(6):646–50. 10.1001/jamadermatol.2024.0468 38452263 PMC10921342

[42] Abeck F Hansen I Wiesenhütter I Schröder F Kött J Schneider SW. Online traffic analysis of direct-to-consumer websites for hair loss treatment and characterization of finasteride patients on a platform in Germany: a potential paradigm shift in the treatment of androgenetic alopecia. Clin Cosmet Investig Dermatol. (2023) Volume 16:937–45. 10.2147/CCID.S400614 PMC10083033 37041819

[43] Young PC Mahajan C Shapiro J Tosti A. Digital health platforms expand access and improve care for male androgenetic alopecia. Int J Dermatol. (2023) 62(2):217–20. 10.1111/ijd.16452 36250302 PMC10092577

[44] Fogel AL Sarin KY. A survey of direct-to-consumer teledermatology services available to US patients: explosive growth, opportunities and controversy. J Telemed Telecare. (2017) 23(1):19–25. 10.1177/1357633X15624044 26729755

[45] Pai VV Pai RB. Artificial intelligence in dermatology and healthcare: an overview. Indian J Dermatol Venereol Leprol. (2021) 87(4):457–67. 10.25259/IJDVL_518_19 34114421

[46] Li C-X Shen C-B Xue K Shen X Jing Y Wang Z-Y. Artificial intelligence in dermatology: past, present, and future. Chin Med J (Engl). (2019) 132(17):2017–20. 10.1097/CM9.0000000000000372 31425274 PMC6793782

[47] Martorell A Martin-Gorgojo A Ríos-Viñuela E Rueda-Carnero JM Alfageme F Taberner R. Artificial intelligence in dermatology: a threat or an opportunity? Actas Dermosifiliogr. (2022) 113(1):30–46. 10.1016/j.ad.2021.07.003 35249709

[48] Bortone G Caro G Ala L Gargano L Rossi A. A new method for the follow-up of patients with alopecia areata. J Clin Med. (2024) 13(13):3901. 10.3390/jcm13133901 38999467 PMC11242085

[49] Torres AE Lim HW. Photobiomodulation for the management of hair loss. Photodermatol Photoimmunol Photomed. (2021) 37(2):91–8. 10.1111/phpp.12649 33377535

[50] Day D McCarthy M Talaber I. Non-ablative er:yAG laser is an effective tool in the treatment arsenal of androgenetic alopecia. J Cosmet Dermatol. (2022) 21(5):2056–63. 10.1111/jocd.14370 34435735 PMC9292628

[51] Ansell DM Kloepper JE Thomason HA Paus R Hardman MJ. Exploring the “hair growth-wound healing connection”: anagen phase promotes wound re-epithelialization. J Invest Dermatol. (2011) 131(2):518–28. 10.1038/jid.2010.291 20927125

[52] Avci P Gupta GK Clark J Wikonkal N Hamblin MR. Low-level laser (light) therapy (LLLT) for treatment of hair loss. Lasers Surg Med. (2014) 46(2):144–51. 10.1002/lsm.22170 23970445 PMC3944668

[53] Faghihi G Mozafarpoor S Asilian A Mokhtari F Esfahani AA Bafandeh B. The effectiveness of adding low-level light therapy to minoxidil 5% solution in the treatment of patients with androgenetic alopecia. Indian J Dermatol Venereol Leprol. (2018) 84(5):547–53. 10.4103/ijdvl.IJDVL_1156_16 30027912

[54] Suchonwanit P Rojhirunsakool S Khunkhet S. A randomized, investigator-blinded, controlled, split-scalp study of the efficacy and safety of a 1550-nm fractional erbium-glass laser, used in combination with topical 5% minoxidil versus 5% minoxidil alone, for the treatment of androgenetic alopecia. Lasers Med Sci. (2019) 34(9):1857–64. 10.1007/s10103-019-02783-8 30982177

[55] Jafari MA Bazgir G Hosseini-Baharanchi FS Jafarzadeh A Goodarzi A. Efficacy and safety of Laser therapy and phototherapy in cicatricial and NonCicatricial alopecia: a systematic review study. Health Sci Rep. (2024) 7(11):e70180. 10.1002/hsr2.70180 39502132 PMC11534645

[56] Nilforoushzadeh MA Fathabadi F Salehi S Heidari A Ghanavati K Najar Nobari N. A systematic review of erbium lasers in the treatment of androgenetic alopecia. Lasers Med Sci. (2025) 40(1):239. 10.1007/s10103-025-04483-y 40402190

[57] Alegre-Sánchez A Jiménez-Gómez N Boixeda P. Laser-Assisted drug delivery. Actas Dermosifiliogr (Engl Ed). (2018) 109(10):858–67. 10.1016/j.ad.2018.07.008 30266385

[58] Rose PT Nusbaum B. Robotic hair restoration. Dermatol Clin. (2014) 32(1):97–107. 10.1016/j.det.2013.09.008 24267426

[59] Daniels G Tamburic S Benini S Randall J Sanderson T Savardi M. Artificial intelligence in hair research: a proof-of-concept study on evaluating hair assembly features. Int J Cosmet Sci. (2021) 43(4):405–18. 10.1111/ics.12706 33848366

[60] Raymond O Shaik J Rypka K Farah RS Bellefeuille G Hordinsky M. Use of artificial intelligence to track platelet-rich plasma treatment outcomes in females with nonscarring alopecia: a case series. JAAD Case Rep. (2024) 47:103–6. 10.1016/j.jdcr.2024.02.037 38699579 PMC11063529

[61] Kuceki G Coppinger AJ Ragi SD Johnson LS Goren A Kalil LL. Enhanced hair regrowth with five monthly sessions of minoxidil-dutasteride-copper peptides tattooing for androgenetic alopecia assessed by artificial intelligence and blinded evaluators. JAAD Int. (2025) 20:38–40. 10.1016/j.jdin.2025.01.012 40225275 PMC11992372

[62] Tuan H Yu L Yin L Lo Sicco K Shapiro J. Prediction of therapeutic outcomes of female pattern hair loss patients based on clinical features with application of artificial intelligence. J Am Acad Dermatol. (2021) 85(6):1622–4. 10.1016/j.jaad.2020.12.035 33352270

[63] Kochmann M Locatis C. Direct to consumer Mobile teledermatology apps: an exploratory study. Telemed J E Health. (2016) 22(8):689–93. 10.1089/tmj.2015.0189 26960113 PMC4982952

[64] McKoy K Halpern S Mutyambizi K. International teledermatology review. Curr Dermatol Rep. (2021) 10(3):55–66. 10.1007/s13671-021-00333-6 34341713 PMC8317676

[65] von Büren J Hansen I Kött J Schröder F Veneroso J Schneider SW. Patient-reported treatment outcomes and safety of direct-to-consumer teledermatology for finasteride treatment in male androgenetic alopecia: a cross-sectional study. Digit Health. (2023) 9:20552076231205740. 10.1177/20552076231205740 37808234 PMC10552455

[66] Maul LV Jahn AS Pamplona GSP Streit M Gantenbein L Müller S. Acceptance of telemedicine compared to in-person consultation from the Providers’ and Users’ perspectives: multicenter, cross-sectional study in dermatology. JMIR Dermatol. (2023) 6:e45384. 10.2196/45384 37582265 PMC10457706

[67] Sharma A Jindal V Singla P Goldust M Mhatre M. Will teledermatology be the silver lining during and after COVID-19? Dermatol Ther. (2020) 33(4):e13643. 10.1111/dth.13643 32441373 PMC7267127

[68] Hindelang M Tizek L Harders C Sommer-Eska L. Hybrid care potential of teledermatology: the importance of linking digital and physical practice and acceptance of online services: a cross-sectional study. Health Sci Rep. (2024) 7(7):e2241. 10.1002/hsr2.2241 38983681 PMC11231929

[69] Abeck F Hansen I Wiesenhütter I Brookman-May SD Sharaf K Kött J. A rejected hypothesis: phenomenon of high treatment adherence in direct-to-consumer teledermatology despite lack of direct physician-patient interaction. Telemed J E Health. (2023) 29(7):1051–6. 10.1089/tmj.2022.0290 36480808

[70] Abeck F Hansen-Abeck I Kött J Garrahy E Schneider SW Von Büren J. Direct-to-Consumer teledermatology for male androgenetic alopecia: narrative review. JMIR Dermatol. (2025) 8:e72704. 10.2196/72704 41418299 PMC12716827

[71] Ahuja S Briggs SM Collier SM. Teledermatology in rural, underserved, and isolated environments: a review. Curr Dermatol Rep. (2022) 11(4):328–35. 10.1007/s13671-022-00377-2 36310767 PMC9589860

[72] Maddukuri S Patel J Lipoff JB. Teledermatology addressing disparities in health care access: a review. Curr Dermatol Rep. (2021) 10(2):40–7. 10.1007/s13671-021-00329-2 33747638 PMC7953516

[73] Ebad SA Alhashmi A Amara M Miled AB Saqib M. Artificial intelligence-based software as a medical device (AI-SaMD): a systematic review. Healthcare (Basel). (2025) 13(7):817. 10.3390/healthcare13070817 40218113 PMC11988595

[74] Bollmeier SG Stevenson E Finnegan P Griggs SK. Direct to consumer telemedicine: is healthcare from home best? Mo Med. (2020) 117(4):303–9. 32848261 PMC7431063

[75] Zhang C Yu Y Shi S Liang M Yang D Sui N. Machine learning guided discovery of superoxide dismutase nanozymes for androgenetic alopecia. Nano Lett. (2022) 22(21):8592–600. 10.1021/acs.nanolett.2c03119 36264822

[76] Ma B Liu D Wang Z Zhang D Jian Y Zhang K. A top-down design approach for generating a peptide PROTAC drug targeting androgen receptor for androgenetic alopecia therapy. J Med Chem. (2024) 67(12):10336–49. 10.1021/acs.jmedchem.4c00828 38836467

[77] Yan P Sun J Zhao Y Deng W Zhang M Li Y. Machine learning-driven optimization of therapeutic substance composition for high-hardness, fast-dissolving microneedles for androgenetic alopecia treatment. ACS Nano. (2025) 19(32):29301–15. 10.1021/acsnano.5c05505 40782084

[78] Naik N Hameed BMZ Shetty DK Swain D Shah M Paul R. Legal and ethical consideration in artificial intelligence in healthcare: who takes responsibility? Front Surg. (2022) 9:862322. 10.3389/fsurg.2022.862322 35360424 PMC8963864

